# ﻿Life history of Papilio (Druryia) meriones Felder & Felder (Lepidoptera, Papilionidae) from Madagascar

**DOI:** 10.3897/zookeys.1239.147822

**Published:** 2025-05-30

**Authors:** Sixun Ge, Lili Ren, Shaoji Hu

**Affiliations:** 1 College of Forestry, Beijing Forestry University, Beijing, China Beijing Forestry University Beijing China; 2 Institute of International Rivers and Eco-security, Yunnan University, Kunming, China Yunnan University Kunming China

**Keywords:** Development, genitalia, immature stages, Papilionidae, swallowtail butterflies

## Abstract

The swallowtail butterflies (Lepidoptera: Papilionidae) have been researched extensively as a model species in biology, ecology, and conservation biology. During a recent expedition to Central and South Madagascar, eggs of a Madagascan-specific swallowtail butterfly, *Papiliomeriones*, were collected. After a successful rearing experiment, the life history of *P.meriones* is described with all developmental stages illustrated.

## ﻿Introduction

The swallowtail butterflies (Lepidoptera: Papilionidae) are one of the most famous and well-studied insect groups, with many species serving as model organisms in evolutionary biology, ecology, genomics, and conservation biology (e.g., Kunte 2009; Kunte et al. 2014; Dupuis and Sperling 2015). The genus *Papilio* Linnaeus, 1758, which includes over 200 known species, is considered the largest genus in the family (Zakharov et al. 2004; Condamine et al. 2012; Nakae 2021). Despite numerous studies carried out on interspecific phylogenetics within the group over the past few decades, it was only in recent years that the classification within the genus was revised (Condamine et al. 2023) due to its vast diversity.

Papilio (Druryia) meriones C. Felder & R. Felder, 1864 is a distinct species found in Madagascar. It was formerly a subspecies of the African mocker swallowtail, *P.dardanus* Yeats, 1776, a well-known polymorphic Batesian mimic that served as a model organism in evolutionary biology (Clarke and Sheppard 1960; Timmermans et al. 2014, 2020). Intriguingly, the closely related species, *P.meriones* has females with “male-like” wing patterns that are monomorphic and non-mimetic. The evolution of the “male-like” female pattern of *P.dardanus* and its two closest relatives, *P.humbloti* Oberthür, 1888 and *P.meriones*, has been a source of speculation, discussion, and ongoing investigation for over 150 years (e.g., Trimen 1869; Poulton 1924; Ford 1936; Bernardi 1963; Vane-Wright 1978; Clarke et al. 1985; Vane-Wright and Smith 1991; Timmermans et al. 2017, 2020). Despite previous studies into the evolution and mimicry of these species, our understanding of their immature stages remains limited. The eggs and larvae of *P.meriones* were obtained during a recent expedition to Central and South Madagascar. After a successful rearing experiment, the morphological characters and duration of each immature stage are reported herein.

## ﻿Material and methods

Eggs and larvae of *P.meriones* were collected in the forest near Tobindranon’i Mantasoa (18.998455°N, 47.874170°E). Shoots and leaves of the host plant (*Citrus* sp.) bearing eggs or larvae were clipped from the host plant and put into plastic boxes. Branches of the host plant with healthy leaves were also collected and sealed in plastic bags.

The rearing experiment was performed without using a climate chamber, and the ambient temperature was approximately 25 °C, with a humidity of 80%. Each individual was observed and recorded each day. Waste and leaf residue were removed from the boxes on a daily basis to keep the environment clean. The size of eggs and body length of each instar larva, prepupa, and pupa were measured.

The immature stages were photographed using an interchangeable-lens digital camera. Measurements and figures of larval head capsules were made using a ZEISS AxioZoom V16 stereomicroscope with a ZEISS Axiocam 503 color camera. Photographs were combined using ZEN 2.3 (blue edition). Final plates were prepared in Adobe Photoshop CC (adobe.com).

The specimen collection has been approved by the government of Madagascar, with the authorization permit number: N°11019/FAC.Sci/D. Specimens used in this study have been deposited in Beijing Forestry University and are available for re-examination by any researcher on request to Si-Xun Ge.

## ﻿Results

### ﻿Developmental stages

**Figure 1.**
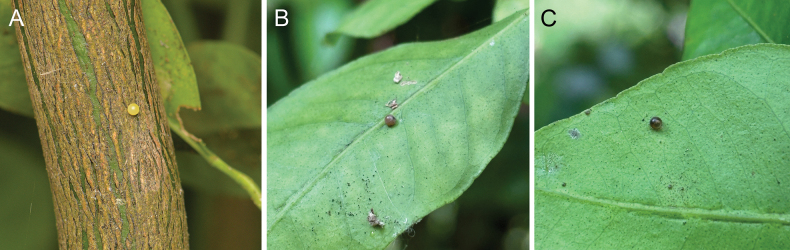
Eggs of *P.meriones***A** freshly laid egg **B** about 2-day-old egg **C** egg about to hatch.

**Eggs.** Female adults laid eggs singly on the upper side or underside of leaves (Fig. 1); sometimes eggs were even laid on the new shoots at the bottom of the plant or on old branches. Eggs spherical, 1.00–1.05 mm in diameter. Surface smooth with small dents, glossy yellowish with pearl sheen (Fig. 1A), gradually turning dark brownish as they develop (Fig. 1B, C). Duration of eggs about 4–6 d.

**Figure 2.**
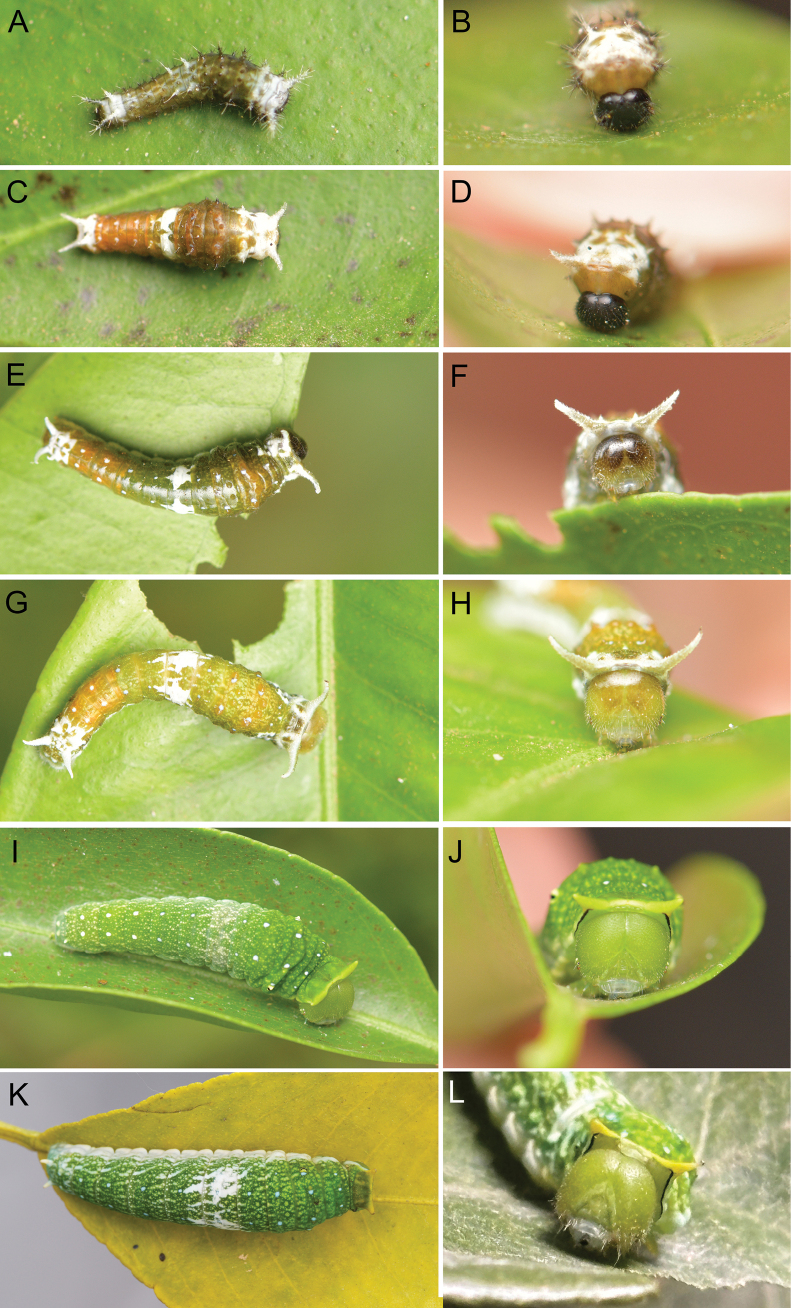
Larvae of *P.meriones***A** 1^st^ instar larva, dorsal view **B** 1^st^ instar larva, head capsule **C** 2^nd^ instar larva, dorsal view **D** 2^nd^ instar larva, head capsule **E** 3^rd^ instar larva, dorsal view **F** 3^rd^ instar larva, head capsule **G** 4^th^ instar larva, dorsal view **H** 4^th^ instar larva, head capsule **I** 5^th^ instar larva (greenish form), dorsal view **J** 5^th^ instar larva (greenish form), head capsule **K** 5^th^ instar larva (whitish-spots form), dorsal view **L** 5^th^ instar larva (whitish-spots form), head capsule.

**Figure 3.**
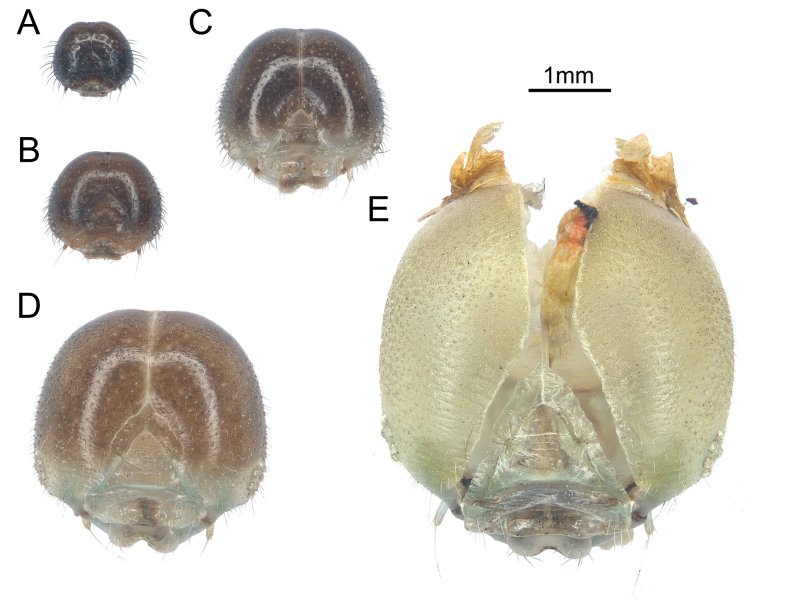
Head capsules of *P.meriones* larvae **A** 1^st^ instar larva **B** 2^nd^ instar larva **C** 3^rd^ instar larva **D** 4^th^ instar larva **E** 5^th^ instar larva.

**Larva.** 1^st^ instar larva (Fig. 2A, B): Maximum width of head capsule 0.92–0.97 mm (Fig. 3A). Body length 5–6 mm. Body olive green with olive or whitish verrucae. Head capsule glossy black. Thoracic verrucae whitish, with the verruca on the 1^st^ thoracic segment extremely elongated. Abdominal verrucae variable, with those on the 1^st^–3^rd^ and 6^th^–8^th^ abdominal segments darkish olive, while those on the 4^th^–5^th^ and 8^th^–9^th^ whitish, verrucae elongated in the last two abdominal segments. All verrucae with long setae. Duration of 1^st^ instar larvae about 4–6 d.

2^nd^ instar larva (Fig. 2C, D): Maximum width of head capsule 1.33–1.39 mm (Fig. 3B). Body length 8–10 mm. Body olive yellow with whitish bands, verrucae olive yellow or whitish. Head capsule glossy black. Thoracic segments and verrucae largely whitish; verruca on the 1^st^ thoracic segment robust and elongated, while those on the 2^nd^–3^rd^ thoracic segments short. The 3^rd^, 8^th^ and 9^th^ abdominal segments whitish. Abdominal verrucae largely small and short, except those on 9^th^ and 10^th^ abdominal segments, which are elongate. All verrucae with long setae. Duration of 2^nd^ instar 5–6 d.

3^rd^ instar larva (Fig. 2 E, F): Maximum width of head capsule 1.98–2.09 mm (Fig. 3C). Body length 9–13 mm. Body olive green to olive yellow with whitish markings. Head capsule glossy dark olive with clypeus area paler. The 1^st^ thoracic verrucae large, elongate into a horn-like shape. Verrucae on 8^th^ and 9^th^ abdominal segments elongate. All elongate verrucae whitish, with rather short setae. Verrucae on other segments rather weak, with a pair of whitish spots on the basal part. The 3^rd^ abdominal segments with a pair of large sub-rhombus whitish spots. In this instar, the larvae evert dark reddish brown osmeteria when disturbed. Duration of 3^rd^ instar 5–6 d.

4^th^ instar larva (Fig. 2G, H): Maximum width of head capsule 2.7–2.87 mm (Fig. 3D). Body length 13–16 mm. Body olive green to olive yellow with whitish markings, whitish spots near the weak verrucae with slightly blueish tone. Head capsule olive yellow on frons area, while olive green on clypeus area. The 1^st^ thoracic verrucae large, elongate into a horn-like shape. Verrucae on the 8^th^ abdominal segment reduced, while on 9^th^ abdominal segment elongate. All elongated verrucae whitish, with setae rather short. The 3^rd^ abdominal segment with a pair of large, irregular whitish spots extending to the 2^nd^ and 4^th^ abdominal segments. Duration of 4^th^ instar 6–8 d.

5^th^ (final) instar larva (Fig. 2I–L): Maximum width of head capsule 4.18–4.45 mm (Fig. 3E). Body length 18.5–45 mm. Body colour largely green with densely scattered pale whitish spots (sometimes with whitish markings), whitish spots near the weak verrucae with blueish tone. Head capsule completely greenish. The first thoracic verrucae on the pronotum reduced, short, and blunt without setae. Verrucae on 8^th^ abdominal segment absent; while on 9^th^ abdominal segment weakened, short and robust. In this instar, the larvae were observed with two different colour forms. The whitish-spots form has a pair of large irregular whitish spots on the 3^rd^ abdominal segment, extending to the 4^th^ abdominal segment (Fig. 2K, L); while the greenish form lacks whitish spots but has a slightly paler 3^rd^ abdominal segment (Fig. 2I, J). Duration of 5^th^ instar 14–16 d.

**Figure 4.**
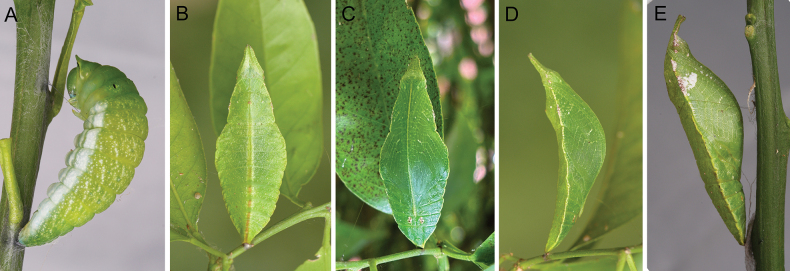
Prepupa and pupa of *P.meriones***A** prepupa **B** greenish form pupa, dorsal view **C** greenish form pupa, ventral view **D** greenish form pupa, lateral view **E** whitish spot form pupa, lateral view.

**Prepupa.** 38–45 mm in length. Body colour light emerald green with semi-transparent tone (Fig. 4A), similar to the 5^th^ instar larva. Duration of prepupa 1–2 d.

**Pupa.** 36–42 mm in length. Body emerald green (Fig. 4B–E). Pupa mimics a curved leaf. Head angular with a pair of emerald green processes, with its apical part close together. Pupae were observed as shown in two different colour forms, corresponding to the colour form of 5^th^ instar larvae. Pupae of the whitish-spots form larvae have a sub-triangular whitish spot near the basal part of the wing, while the greenish form lacks the whitish spot. Duration of pupa 13–16 d.

**Figure 5.**
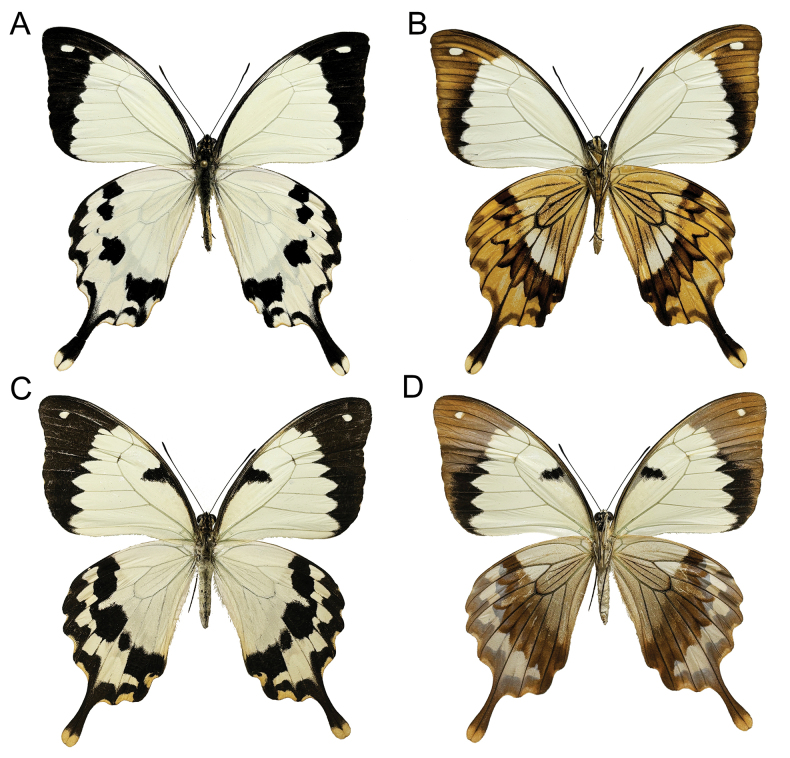
Adults of *P.meriones***A** male upperside **B** male underside **C** female upperside **D** female underside.

**Adults.** Male (Fig. 5A, B): Length of fore-wing about 50 mm. Upperside (Fig. 5A): Fore- and hind-wings largely whitish. Fore-wing with outer margin blackish, broadening at the apex. Sub-apical area with an oval whitish spot. Hind-wing with blackish outer margin, extending from space 5 to space 8. Spaces 2–4 with crescent-shaped blackish spots near the outer margin. Spaces 1b, 2, 4, 6 and 7 with sub-rectangular blackish spots. Tornus with a blackish spot. Tails blackish, with tips pale creamy whitish. Underside similar to the upperside (Fig. 5B), but with all blackish markings pale brownish. Hind-wing with brownish lines well-developed in all spaces, forming a radial pattern.

**Figure 6.**
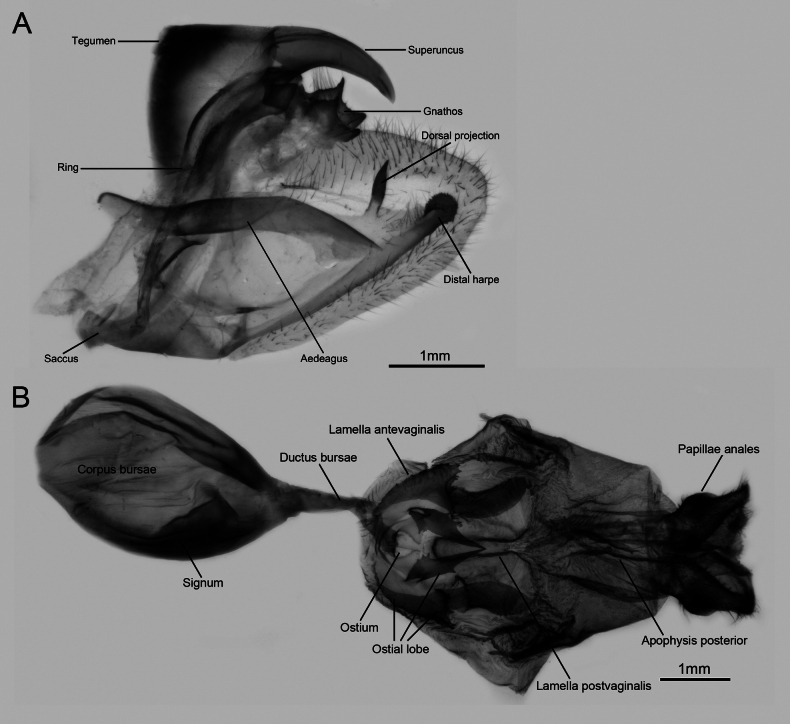
Genitalia of *P.meriones***A** male genitalia **B** female genitalia.

Male genitalia (Fig. 6A): Highly sclerotised. Ring slightly wavy in the upper part, with distinctly developed bicornate gnathos on the tip; tegumen broad; superuncus broad at the base, slightly narrowed towards the tip and curved ventrally in lateral view; saccus flat, more or less reduced. Valve sub-triangular shaped, slightly elongate with its distal part blunt; the distal harpe strongly sclerotised, toothed disc-shaped; the dorsal projection digit-shaped with an acutely pointed tip. Aedeagus robust, more or less wavy.

Female (Fig. 5C, D): Similar to male, but fore-wing with a blackish marking in the discal cell. On the upperside, it extends from the basal of vein Sc to the sub-apical part of the discal cell (Fig. 5C), while on the underside it is developed as an oblique sub-rectangular dark brownish spot in the median part of the discal cell (Fig. 5D).

Female genitalia (Fig. 6B): Apophysis posterior short and slender; lamella postvaginalis lobe-shaped with inner distal edge extending to the centre. Ostial lobe strongly sclerotised; inner part of the ostial lobe multi-toothed sub-triangular or sub-quadrangular shaped, with its apical tip rather elongate; outer part of the ostial lobe flange-shaped, with its outer margin multi-dentate and with a prominent protrusion in the sub-median part.

## ﻿Discussion

The African mocker swallowtail, *Papiliodardanus*, is a well-known example of a polymorphic Batesian mimic. It has played an important part in the debate over the evolution of phenotypic variation. Studies on the mimicry of the species date back over a century (Trimen 1869; Poulton 1924; Ford 1936). However, in terms of systematics and taxonomy, the first molecular approach involving the subgenus Druryia was only recently carried out (Condamine *et al*. 2023); it supported *P.meriones* as a distinct species rather than a subspecies of *P.dardanus*.

**Figure 7.**
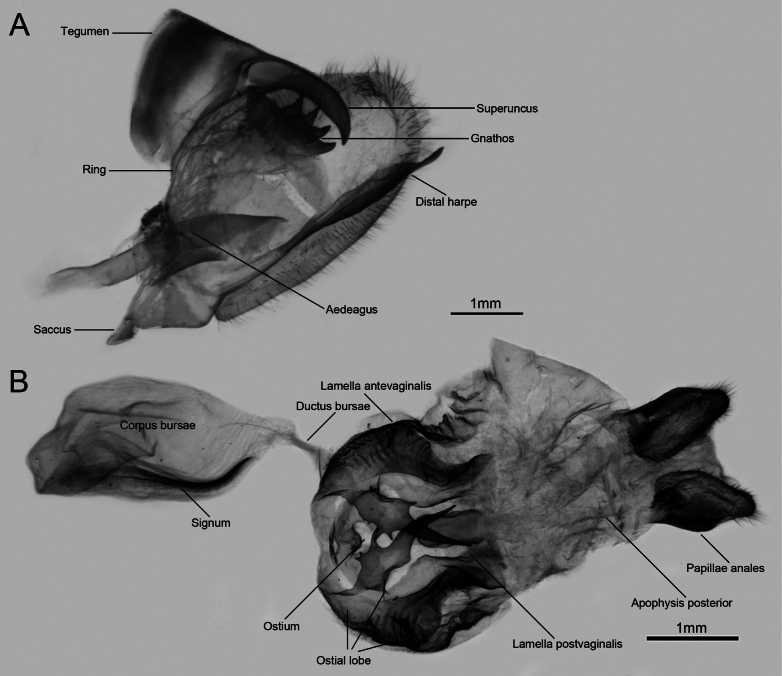
Genitalia of *P.d.dardanus***A** male genitalia **B** female genitalia.

Morphological data from immature stages are extremely important in taxonomy, systematics, and evolutionary biology in Lepidoptera. Our study describes the life history of a Madagascan endemic species, *P.meriones*, with a focus on the morphology of immature stages. As closely related species, *P.dardanus*, *P.meriones*, and *P.humbloti* exhibit distinct differences in their male genitalia, particularly in the dorsal projection on the inner side of the valve (Carpenter 1948; this study). *Papiliomeriones* possesses a strongly sclerotized distal harpe, apically toothed and disc-shaped, with a sclerotized digit-shaped dorsal projection (Fig. 6A). In contrast, the distal harpe of *P.d.dardanus* is highly elongated beyond the valve (Fig. 7A), laminar in structure, and lacks a dorsal projection. Specimens from the eastern Madagascan populations (including *P.d.tibullus*, *P.d.antinorii*, *P.d.byatti*, and *P.meriones*) always display a digit-shaped dorsal projection with an acutely pointed tip. In contrast, western specimens (*P.d.dardanus*, *P.d.meseres* and *P.humbloti*) show a highly reduced or vestigial dorsal projection. In the female genitalia, *P.meriones* and *P.d.dardanus* also exhibit distinct differences (Figs 6B, 7B), primarily manifested in the morphology of the ostial lobe.

In terms of immature stages, these closely related species exhibit subtle yet diagnostically significant divergences (Williams 2023, this study): The 1^st^ instar larvae: (1) *P.dardanuscenea* Stoll, 1790 presents a predominantly blackish-brown coloration, contrasting with much paler *P.meriones* larvae; (2) the W-shaped marking on the 6^th^ segment (3^rd^ abdominal segment) of *P.dardanuscenea* larvae is absent in *P.meriones*; and(3) *P.meriones* larvae have the 4^th^ and 5^th^ abdominal segments whitish, while in *P.dardanuscenea* uniformly darkish. The 2^nd^ instar larvae show no discernible interspecific differentiation, while divergences re-emerge in the 3^rd^ instar: (1) *P.dardanuscenea* larvae develop double rows of small blue tubercles restricted to the 3^rd^ thoracic and 1^st^, 4^th^, 6^th^ abdominal segments, whereas *P.meriones* exhibit continuous tubercle rows from the 2^nd^ thoracic segment to the 7^th^ abdominal segment (excluding the 3^rd^ abdominal segment); (2) The white marking on the 3^rd^ abdominal segment is expanded in *P.dardanuscenea* larvae, while reduced to the segment interior in *P.meriones*; and (3) The head capsule of *P.dardanuscenea* larvae is greenish, whereas that of *P.meriones* is glossy dark olive with pale clypeus. Later instars and pupae demonstrate morphological convergence between species; however, the whitish spot form of 5^th^ larvae and pupa characterized in *P.meriones* are not reported in *P.dardanuscenea* descriptions.

Madagascar is a hotspot for evolutionary biological research, and its fauna is widely regarded as unique. Intriguingly, the closely related species of the model of mimetic *P.dardanus* were also found in Madagascar (e.g., *Amaurisnossima* (Ward, 1870)), but *P.meriones* did not evolve Batesian mimicry in the same way that *P.dardanus* did. In addition, *P.dardanusantinorii* Oberthür, 1883 from Ethiopia, as well as another closely related species, *P.humbloti* endemic in Comoro Is, are also non-mimetic.

## ﻿Additional information

### Author ORCIDs

Sixun Ge https://orcid.org/0000-0003-3769-1530

### Data availability

All of the data that support the findings of this study are available in the main text.
